# Immediate Caregiving Environment of Young Children with Autism: Findings from the U.S. National Survey of Children’s Health

**DOI:** 10.3390/ijerph21010012

**Published:** 2023-12-21

**Authors:** Hong Li, Teresa Dodd-Butera, Margaret L. Beaman, Rebecca Burtea

**Affiliations:** 1 Department of Doctoral Studies, Institute of Health Research, School of Nursing, Azusa Pacific University, 606 E Huntington Dr., Room 235, Monrovia, CA 91016, USA; 2 Departments of Public Health and Doctoral Studies, Institute of Health Research, School of Nursing, Azusa Pacific University, San Diego, CA 92108, USA; tbutera@apu.edu; 3 Department of Nursing, California State University, San Bernardino, CA 92407, USA; 4 Department of Mathematics, Physics, and Statistics, Azusa Pacific University, Azusa, CA 91702, USA; rebeccaburtea@gmail.com

**Keywords:** autism spectrum disorder, parental emotional support, parent–child interactions, children with non-special health care needs

## Abstract

Autism spectrum disorder (ASD) is a complex neurodevelopmental disability that negatively affects children’s learning, motor behavior, social communication, and interaction. It was estimated that, in 2020, 1 in 36 children aged 8 years in the United States had ASD. Caring for children with ASD might exert significant psychological and emotional distress on parents. Receiving parental emotional support and fostering positive parent–child interactions at home have been identified as beneficial for the immediate caregiving environment for children with ASD. The current secondary analysis of the 2019–2020 National Survey of Children’s Health examined parent–child interactions and accessible sources of emotional support for parents caring for 3–5-year-old children diagnosed with ASD (*N* = 243). Children with the following characteristics had higher odds of having ASD: male gender; having no private insurance or uninsured; and having less than excellent general health. Among parents, higher odds of caring for children with ASD were associated with accessing emotional support from various sources, especially from healthcare professionals and peers, and spending more time telling stories and/or singing to their children. Given these significant health disparities, educational interventions and strategies are needed to foster a positive home caregiving environment for young children with ASD, including equitable access to parent resources.

## 1. Introduction

Autism spectrum disorder (ASD) is a complex neurodevelopmental disability that can cause a wide range of impairments in the social, learning, communication, and functioning aspects of people’s lives [1]. Although ASD symptoms are commonly identified in early childhood (ages 3–4), ASD affects people from infancy through adulthood [2]. It is estimated that about 1 in 100 children around the world has ASD, although the prevalence of ASD in many low- and middle-income countries is under-reported or under-represented in research studies [3]. In the United States, the prevalence of ASD in childhood rose steadily between 1997 and 2017 [2], and between 2017 and 2020 [4]. More specifically, a CDC report estimated that in 2020, about 1 in 36 8-year-old children in the United States had an ASD diagnosis [5]. The overall prevalence is similar across racial and ethnic groups, but the rate is 3.8 times as prevalent among boys (1 in 23) than girls (1 in 88) [5].

According to the American Psychiatric Association [6], people with ASD experience persistent deficits in social communication and interaction. These deficits are manifested in social–emotional reciprocity, nonverbal communicative behaviors, and/or difficulties in developing, maintaining, and understanding relationships. People with ASD also display restricted and/or repetitive patterns of behavior or interests, including stereotyped or repetitive motor movements, inflexible adherence to routines, fixated strong interests in unusual objects, or hyper- or hypo-reactivity to sensory input [7]. These clinical manifestations can occur in a continuum of severity, ranging from mild to severe, and can be presented in diverse ways across age groups, gender groups, and developmental stages [7]. This clinical continuum may impact the range and severity of experiences and emotional needs of parents and families of children diagnosed with ASD [8,9].

Given the challenging problems that children with ASD experience in social interaction, communication, and information processing, childcare places significant daily and long-term demands on parental caregivers [10]. The potential range of caregiving responsibilities of parents of children with ASD is extensive, from interpreting behaviors, monitoring conditions, providing hands-on care, developing care routines, managing and maintaining health in the home, making decisions, and time management, to problem-solving [11]. To achieve effective caregiving, parents often need to reorganize their daily lives to support their child’s specific needs, by reducing work hours or even leaving the workforce to become full-time caregivers [12]. These caregiving-related stressors and burdens negatively affect parents’ mental health-related well-being [13,14,15,16,17,18,19,20] and physical health [21,22]. Parents of children with ASD also face system-level challenges due to difficulties in accessing, navigating, and participating in professional services. These challenges place a great amount of mental distress on parents [23] which can be detrimental to caregiving of children with ASD. For example, parents who have their own mental health difficulties (e.g., depression or anxiety) may face additional challenges to ensure effective and compassionate care for their children [24].

Empirical evidence suggests that receiving emotional support from family members, peers, and healthcare providers has many benefits, including improving parental quality of life [25], reducing the risk of parental depression [26], helping parents feel more hopeful and respected [16], and increasing parental resilience [27]. Accessing and receiving social and emotional support are important strategies to help parents adapt, adjust, and handle challenging situations, which are crucial to the quality of the caregiving environment of children with ASD. Types of services often utilized by parents caring for children with ASD include mental health counseling, speech/language therapy, occupational therapy, physical therapy, and social skills training [28]. Although formal and informal support is beneficial in decreasing stress for rural caregivers of children with ASD [29], parents in rural areas reported less access to resources and services compared with those in urban areas [30]. Also, support service use and need vary greatly among toddlers, school-aged children, adolescents, and adults with ASD [31]. Other suggested facilitators for effective caregiving include positive parent–child interactions and family activities [32]. Family connectedness may be impacted by children’s ASD conditions. For example, a recent study reported that children with ASD were more likely to have no family meals each week than children without ASD [33]. In addition, an increased severity of ASD adversely impacted the quality of parent–child interactions, such as communication, emotional expression, and responsivity [34].

The current study specifically adds to the body of knowledge regarding stressors on families with children, aged 3–5 years old, diagnosed with ASD. Sources of parental emotional support and parent–child interactions in the home are critical elements of care for well-being in families of young children with ASD and provide information for formulating the basis of effective, evidence-based interventions. To gain further understanding of the accessible sources of emotional support for parents, the quality of their interactions with children at home among families of children with ASD and children with no special health care needs (non-CSHCN) should be assessed and compared. For consistency, the same abbreviations (i.e., non-CSHCN) are used in this article as suggested by the NSCH study.

The approach for this study includes a broad definition of environmental factors in relation to ASD in children. To further operationalize this concept, environmental factors of children with ASD include the immediate conditions in which families experiencing ASD live and provide day-to-day care in relation to children’s physical, emotional, social, and overall well-being [11]. This comprehensive definition of environmental factors is based on the underlying assumption that primary caregiving of children with ASD might be both satisfying and stressful for the caregivers. Therefore, the significant emotional, family, and social stress involved in caring for children with ASD has the potential to be detrimental to the immediate caregiving environment of these children. Reviewing family support, along with emotional and social stressors, contributes to an accurate depiction of the caregiving environment for families with children experiencing ASD.

## 2. Purpose and Aims

The overall objective of this retrospective, cross-sectional secondary data analysis study was to describe and compare three aspects of families with 3–5-year-old children diagnosed with autism spectrum disorder (ASD) and families who are not caring for children with special health care needs (CSHCN): sociodemographic characteristics, sources of parental emotional support, and parent–child interactions in their homes. Specifically, this study aimed to answer the following research questions: (1) Are there differences in parent–child interactions at home and parental emotional support when comparing families with children 3–5 years old with ASD and non-CSHCN? (2) Are there differences in parent–child interactions at home and parental emotional support when comparing families with children ages 3–5 with mild, moderate, or severe ASD? (3) Is there an association between sources of emotional support for parents caring for children 3–5 years old and their child’s ASD vs. non-CSHCN status? and (4) Is there an association between parent–child interactions in the homes of families of children 3–5 years old and their child’s ASD vs. non-CSHCN status? The variations in parental emotional support and parent–child interactions across ASD-related health conditions will inform family nurses, social workers, community leaders, and faith leaders who design effective interventions for fostering a positive home caregiving environment for young children with ASD.

## 3. Materials and Methods

### 3.1. Study Population, Setting, and Data

The 2019–2020 National Survey of Children’s Health (NSCH) data set was utilized as the principal data source. The NSCH annually collects information related to the physical health and emotional well-being of noninstitutionalized children from birth to 17 years in the U.S. [35]. The information includes access to and use of health care, family interactions, parental health, school and after-school experiences, and neighborhood characteristics. The survey is completed by adult participants who have children aged 0–17 living at the sampled residential address and are eligible for the study. The survey utilizes three age-based topical questionnaires tailored to children aged 0–5, 6–11, and 12–17. Detailed descriptions of the NSCH survey design, procedures, and implementation processes are reported extensively elsewhere [36].

The NSCH oversamples children with special health care needs (CSHCN), including those with ASD [37]. Using an address-based sampling approach, the 2019–2020 NSCH produced national- and state-representative estimates of children’s health, with a combined number of 420,000 households recruited, 87,303 households screened, and 72,210 topical questionnaires completed for children aged 0–17 years [37]. The overall weighted response rate was 42.4% for both 2019 and 2020. The study sample in the present study comprised 243 children reported to have ASD and 9798 non-CSHCN in the 3–5 years age group, as identified in the 2019–2020 NSCH data set. 

### 3.2. Data Retrieval and Ethical Considerations

The research team submitted the data user agreement for the 2019–2020 NSCH data from the Data Resource Center for Child and Adolescent Health (DRC). The DRC granted the team permission to use the data and provided a downloadable link. Since the DRC provides de-identified case-level NSCH data for public use [35], human subjects were not involved in the design, data extraction, data analysis, or creation of this research report. Therefore, the Institutional Review Board (IRB) at the lead researcher’s institution deemed this retrospective research study as exempt from full or expedited review.

### 3.3. Measures

#### 3.3.1. Children’s Sociodemographic Characteristics

Children’s age was measured as a continuous variable in years (i.e., 3, 4, 5). Children’s gender was measured using standard NSCH categories (i.e., female, male). Children’s race and ethnicity were reported in the following four categories: Hispanic, White non-Hispanic, Black non-Hispanic, and Other/Multiracial non-Hispanic. For this study, due to small sample sizes in select racial groups, a dichotomous variable was created for race as either “White, non-Hispanic” (67.76%) or “Others” (32.54%). Children’s health insurance coverage at the time of the survey was reported in the following categories, “public health insurance only”, “private health insurance only”, “public and private insurance”, and “uninsured”. For this study, a median split was used to categorize the type of health insurance as either “have private insurance” (74.54%) or “do not have private insurance or uninsured” (25.35%). These recoding approaches are consistent with prior studies using NSCH data [38,39].

#### 3.3.2. Parental Socioeconomic Status

Parental highest level of education was reported as either “with a college degree or higher”, “some college or technical school”, “high school or GED”, or “less than high school education”. For data analysis in the present study, the parental highest level of education was coded as either “with a college degree or higher” (65.72%) or “do not have a college degree” (34.57%). This recoding approach is consistent with prior studies using NSCH data [39]. Household income level was assessed by the combined family income reported in NSCH as a percentage of the federal poverty level (FPL) during the corresponding year in the following categories: 0–99% FPL, 100–199% FPL, 200–399% FPL, and 400% FPL or greater. For this study, reported household income was divided into two categories using the median split, either “below 400% FPL” (59.96%) or “400% FPL or greater” (40.04%).

#### 3.3.3. Children’s General Health

This variable was informed by parents’ or other caregivers’ responses to the question “In general, how would you describe the child’s health?” on a 5-point Likert scale ranging from 1 “excellent” to 5 “poor”. For this study, this variable was reverse coded so that a rating of 1 indicates poor health while 5 indicates excellent health [40]. Moreover, for this study, a median split was used to create a dichotomous variable for children’s general health as either “excellent” (78.00%) or “less than excellent” (22.00%). This recoding approach is similar to prior studies using NSCH data [41].

#### 3.3.4. Children’s ASD-related Health Conditions

Children’s special health care needs status was informed by parents or other caregivers in response to a five-item screener: (1) use or need of prescription medication; (2) above average use or need of medical, mental health, or education services; (3) functional limitations compared with others of the same age; (4) use or need of specialized therapies; and (5) treatment or counseling for emotional or developmental problems [37]. Children reported to have experienced none of the five needs were identified as non-CSHCN in the NSCH dataset. Children’s ASD status was informed by parents’ or other caregivers’ response to the following question “Does this child currently have Autism or Autism Spectrum Disorder (ASD)?” Subsequently, children’s ASD condition severity was reported as 1, indicating mild, 2, indicating moderate, and 3, indicating severe ASD. The above CSHCN screener questions do not permit any diagnosis-specific information, as the intention of the NSCH is to identify a wide range of health consequences a child experiences due to a health condition rather than on the presence of a specific clinical diagnosis [42].

#### 3.3.5. Parental Emotional Support Accessibility (PESA)

The accessibility of emotional support was measured by the question “During the past 12 months was there someone that you could turn to for day-to-day emotional support with parenting or raising children?” An answer of “yes” indicated having access to emotional support. Subsequently, resources that parents had accessed for emotional support were assessed by responses to the following question with seven options: “If yes, did you receive emotional support from (a) spouse or domestic partners? (b) other family member or close friend? (c) health care provider? (d) place of worship or religious leader? (e) support or advocacy group related to specific health condition? (f) peer support group? (g) counselor or other mental health professional?” An answer of “yes” to each of the seven sources indicates having access to emotional support from the corresponding source. Therefore, the composite score of PESA, computed by adding the scores from all sources, ranged from 0 to 7. In addition, a median split of the PESA composite score was used to categorize high (58.68%) vs. low (41.32%) levels of overall access to parental emotional support. This approach is similar to the procedures in prior studies using NSCH data [39].

For this study, the seven sources were collapsed into four categories to reflect similar types of support: “family/friends” (i.e., sources a and b), “faith community” (i.e., source d), “healthcare professionals” (i.e., sources c and g), and “advocacy and peer support groups” (i.e., sources e and f). Scores of all four categories ranged from 0 to 2, except for the category of “faith community” which ranged from 0 to 1. Cronbach’s alpha for this 4-category scale was 0.59 in children with ASD and 0.53 in non-CSHCN. The alphas were not expected to be high due to a low number of items in each category [39]. For logistic regression analysis, a dichotomous variable was created for each of the four categories, with 1 indicating “yes” and 0 indicating “no” support received from the corresponding category of sources.

#### 3.3.6. Parent–Child Interactions (PCIs)

This index was created from three NSCH-reported indicators of parent–child interactions and bonding activities, including “parents’ time spent on reading to the children during the past week”, “parents’ time spent on telling stories and/or singing to the children during the past week”, and “during the past week, how many days did all the family members who live in the household eat a meal together?” Frequencies of the above three activities were reported on a 4-point Likert scale ranging from 1 “0 days” to 4 “every day”. A composite PCI score was produced by summing the responses to the aforementioned three parent interaction questions, resulting in a possible total score of 3 to 12. This approach is consistent with prior studies using NSCH data [43]. In the present study, the Cronbach alpha of the three items chosen to measure positive PCIs was 0.63 in children with ASD and 0.58 in non-CSHCN. For logistic regression analysis, a dichotomous variable was created for each of the three PCIs using median splits, with 1 indicating “high” and 0 indicating “low” reported frequencies in the corresponding type of parent–child interaction. A median split was also used to categorize high (54.77%) vs. low (45.23%) levels of overall PCIs.

### 3.4. Statistical Analysis

Descriptive analyses were conducted to illustrate key sociodemographic characteristics of children’s age, gender, race/ethnicity, and ASD status in the 3–5 years old age group. The distribution of children’s health insurance status, family income level, and parents’ educational level in children with ASD and non-CSHCN also were described. For this study, a proportional median split was used to represent the natural patterns of the responses in the raw NSCH data for each study variable. Considering skewed sample sizes of the two groups of children with ASD and non-CSHCN and unequal variances, Welch’s *t*-tests were used to determine significant differences in sociodemographic characteristics, children’s general health, parental emotional support, and parent–child interactions of the two children groups. These differences were further stratified by children’s gender. One-way MANOVA and Wilcoxon signed rank tests were employed to verify differences across children’s special health care needs conditions and ASD severity levels. Last, multivariable logistic regression was performed to estimate the association between parental emotional support, parent–child interactions, and children’s ASD vs. non-CSHCN status, adjusting for children and family sociodemographic factors. The range of variance inflation factors (VIF) levels of 1.05 to 1.68 indicated no multicollinearity among predictor variables. All analyses were conducted in JMP Pro (Version 16.1.0). The two-tailed alpha level for statistical significance was set at 0.05.

## 4. Results

### 4.1. NSCH Reported Child and Family Demographics by Children’s ASD Status

Between 2019 and 2020, U.S. parents completed the NSCH for a total of 10,565 children aged 3 to 5 years. Among these children, 9798 (92.74%) were non-CSHCN and 243 (2.30%) were reported to have a diagnosis of ASD. Among children with ASD, 74.49% were male compared to 49.56% of the non-CSHCN, *t* = 8.75, *p* < 0.0001. Compared with children with ASD, more non-CSHCN were Non-Hispanic White (*M* = 0.68 vs. 0.58, *t* = 2.89, *p* = 0.04), had private insurance (*M* = 0.25 vs. 0.44, *t* = 5.74, *p* < 0.0001), and had better general health (*M* = 4.77 vs. 4.07, *t* = 11.76, *p* < 0.0001). In addition, parents of non-CSHCN had a higher household income (*M* = 0.40 vs. 0.26, *t* = 4.88, *p* < 0.0001) and higher education (*M* = 0.87 vs. 0.79, *t* = 2.87, *p* = 0.005) than parents of children with ASD.

One-way MANOVA did not identify any significant differences in child and family demographics across different ASD severity levels, *p* > 0.05. ANOVA analyses comparing the general health of children with ASD by their levels of ASD severity revealed significant differences, *H*(3) = 11.57, *p* = 0.009. Children with mild ASD were reported to have better general health than children with moderate ASD, *M* = 4.30 vs. 3.90, *p* = 0.0008. See Table 1 for frequency distributions of child and family sociodemographic characteristics across children’s ASD status and severity groups. See Table 2 for means and standard deviations of child and family sociodemographic characteristics across children’s ASD status and severity groups.

Child and family sociodemographic factors were significantly associated with children’s ASD (vs. non-CSHCN) status (chi-square = 259.11, *df* = 7, *p* < 0.001). Logistic regression further identified significant predictors of ASD diagnosis: children’s gender (OR = 2.91, 95% CI = 2.15–3.92), insurance type (OR = 1.66, 95% = 1.22–2.27), and general health (OR = 0.18, 95% = 0.14–0.24). Compared with girls, boys had 191% higher odds of being diagnosed with ASD. Children who had no private insurance or were uninsured (vs. private insurance) had 66% higher odds of being diagnosed with ASD. Children who were reported to have excellent general health had 82% lower odds of being diagnosed with ASD.

### 4.2. Parental Emotional Support by Children’s ASD Status

On average, parents caring for children with ASD had more sources of emotional support (*M* = 2.54, *SD* = 1.78) than those caring for non-CSHCN (*M* = 2.29, *SD* = 1.41), *t* = 2.17, *p* = 0.03. More specifically, compared with parents of non-CSHCN, parents of children with ASD reported higher accessibility of emotional support from health professionals (*M* = 0.56 vs. 0.33, *t* = 4.89, *p* < 0.0001) and from advocacy and peer support groups (*M* = 0.35 vs. 0.17, *t* = 4.06, *p* < 0.0001). However, parents of children with ASD reported less access to emotional support from family and friends than parents of non-CSHCN (*M* = 1.42 vs. 1.57, *t* = 2.68, *p* = 0.008). See Table 3 for descriptive statistics of parental emotional support across children’s ASD status and severity groups.

When all child and family demographic covariates were controlled, logistic regression identified the total PESA score as a significant predictor of parental odds of caring for a child with ASD (vs. non-CSHCN), OR = 1.85, 95% CI = 1.41–2.44. Therefore, parents who accessed a higher level of emotional support had 85% higher odds of caring for children with ASD (vs. non-CSHCN). When the four PESA categories were included simultaneously in the logistic regression model, healthcare professionals (OR = 2.10, 95% CI = 1.53–2.86) and advocacy and peer support groups (OR = 1.66, 95% CI = 1.18–2.33) remained significant predictors of an ASD diagnosis. Parents who had access to healthcare professionals for emotional support had 110% higher odds of caring for children with ASD. Likewise, those who had access to advocacy and peer support groups had 66% higher odds of caring for children with ASD.

Among parents of children with ASD, their total PESA varied across children’s ASD severity levels, *H*(3) = 10.34, *p* = 0.02. Parents of children with moderate ASD had higher accessibility to overall emotional support (*M* = 2.84, *SD* = 1.73) than parents of children with mild ASD (*M* = 2.29, *SD* = 2.84) and severe ASD (*M* = 1.95, *SD* = 2.15), *p* = 0.02. More specifically, parents of children with different severity levels of ASD had varied access to health professionals for emotional support, *H*(3) = 13.37, *p* = 0.004. Parents of children with moderate ASD reported higher access to health professionals (*M* = 0.68, *SD* = 0.72) than parents of children with mild ASD (*M* = 0.42, *SD* = 0.67), *p* = 0.03.

### 4.3. Parent–Child Interactions by Children’s ASD Status

The results of Welch’s *t*-test revealed that children with ASD (*M* = 9.39, *SD* = 2.17) and non-CSHCN (*M* = 9.57, *SD* = 1.95) had the same level of overall interactions with their parents at home (*t* = 1.25, *p* = 0.21). When examining the individual PCI indicators, children with ASD (vs. non-CSHCN) had fewer days in a week sharing meals with family members (*M* = 3.22 vs. 3.41, *t* = 3.04, *p* = 0.003). The reported total positive PCIs or the individual PCI indicators did not vary across children’s three ASD severity levels. See Table 4 for descriptive statistics of PCIs across children’s ASD status and severity groups.

When all child and family demographic covariates were controlled, the total PCI score was not associated with the odds of caring for a child diagnosed with ASD (vs. non-CSHCN), *p* > 0.05. When examining individual PCI indicators, time spent telling stories and/or singing to the child remained a statistically significant predictor of an ASD diagnosis, OR = 2.21, 95% CI = 1.61–3.03. Parents who spent more time telling stories and/or singing to their children had 121% higher odds of caring for children with ASD (vs. non-CSHCN).

## 5. Discussion

To examine the immediate caregiving environment of children with ASD, the investigators applied a broad definition of the environment, i.e., the surroundings or conditions that affect the behavior and development of a person [44]. Therefore, in the current study, socioeconomic factors, parental emotional support, and conditions of the home and family environment were examined in the context of parent–child interactions, as factors for consideration in children with ASD.

### 5.1. Child and Family Demographics

This analysis of the NSCH data identified a higher percentage of boys among children with ASD (74.49%) than among non-CSHCN (49.56%). This agrees with the Autism Developmental Disabilities Monitoring (ADDM) Network which reported a higher percentage of boys than girls identified with ASD [45]. The ADDM Network indicates that for every girl identified with ASD, boys were nearly four times as likely to be identified with the condition [45]. A systematic review and meta-analysis reported that the male-to-female ratio for diagnosis of ASD was closer to 3:1 [46]. Loomes and colleagues [46] further proposed a diagnostic gender bias for females meeting the criteria for ASD, who were disproportionately at risk for not receiving a clinical diagnosis.

In terms of race and ethnicity, findings from the NSCH data in this study indicated that non-CSHCN were more likely to be non-Hispanic White. This supports the key findings of the ADDM Network which report that Black, Hispanic, and Asian or Pacific Islander children were more likely to be identified with ASD than White children [45]. In the current study, compared with children with ASD, non-CSHCN had a higher rate of private insurance, higher household income, and parents with higher levels of education. In contrast, using the CDC Social Vulnerability Index (SVI) which identifies communities that might need support in emergency response and disaster recovery efforts, Patrick et al. [47] found that the overall prevalence of ASD was higher in areas of higher (vs. lower) socioeconomic status. Arguably, such variations in estimated prevalence by key sociodemographic factors reflect the influences of varying biological and/or environmental etiological factors linked with ASD, as well as health disparities associated with racial inequities, stigma, and systemic barriers to healthcare [3].

### 5.2. Emotional Support

In the current study, parents caring for children with ASD reported a higher level of emotional support from healthcare professionals and advocacy and peer support groups than parents with non-CSHCN. However, parents of children with ASD reported less emotional support from family than those of non-CSHCN. In comparison, there is evidence in the literature suggesting a lack of perceived emotional support, a lack of accessible family supportive resources, and a struggle in asking for help among parents with CSHCN, especially with children with ASD [38,48,49]. The lack of emotional support despite the great need for emotional support is among the key barriers to parental mental health well-being [50] and to parents providing effective care for children with ASD [51].

In addition, children’s ASD severity moderated the amount of emotional support their parents received. Parents caring for children with moderate ASD reported significantly higher accessibility to overall emotional support than those of children with mild and severe ASD. This was especially true for reported parental access to health professionals for emotional support. In children, moderate ASD is diagnosed earlier than mild and severe forms of ASD [52]. Thus, it is postulated that the timing of ASD diagnosis may have influenced the reported parental access to emotional support from healthcare professionals. The relationship between parental access to emotional support and children’s ASD severity warrants further investigation.

### 5.3. Parent–Child Interactions at Home

Social communication and meaningful interaction can be central to the challenges facing parents and children experiencing ASD [53,54]. The current study identified that children with ASD (vs. non-CSHCN) had fewer days in a week sharing meals with family members, although no significant differences in the overall parent–child interactions in the home between the two groups were found. Further, levels of parent–child interaction did not vary across the three ASD severity levels. Previous evidence indicates that 2 to 4 year olds with ASD spend less time in coordinated joint engagement [55] that requires coordination of eye contact and socialized interaction including both people and objects. Campbell et al. [56] studied toddlers and found that decreased levels of social engagement were associated with symptom severity within high-risk groups for ASD. In the current study, parents caring for children with ASD spent more time telling stories and/or singing to their children than those of non-CSHCN. Both a recent systematic review of clinical trials [57] and a synthesis of qualitative studies [58] suggested that this type of parent–child interaction is beneficial for autistic children by improving their word reading experiences, quality of life, and even total autism severity.

### 5.4. Study Limitations

The current study is limited by the reliance on self-reported information from a single survey source and limited to two years of cross-sectional data. Although various sampling techniques were utilized to ensure representative samples, the NSCH surveys do not necessarily represent all children with ASD or CSHCN in the U.S. Also, children’s data in NSCH relied on reports from parents or other caregivers. The national survey did not provide an opportunity for parents or caregivers to further elaborate on their responses. This parent proxy report may suffer from recall and social desirability biases, especially when parents respond to children’s general health [59] and parent–child interaction-related questions [60]. Moreover, it is important to note that in the context of the current study sample, children’s ASD status and severity were self-reported by participants without clinical verification. This data collection method may have an impact on the validity and interpretations of the study findings. The impact of the clinical continuum of symptoms and presenting parental stress warrants further study, using varied methods of study to uncover differences based on the level of ASD diagnosis. Also, there are likely to be other types of interactions that could be good measures of parent–child interactions at home, such as children’s free and pretend play with a parent [56]. Finally, several study variables were collapsed into fewer categories for data analysis. This collapsing of data might have resulted in lost information that would provide more precise results for certain children groups [61]. Despite these limitations, the findings of the current study remain valuable to the literature as they reflect parental emotional support needs and how they interact with their children with ASD in the home environment.

### 5.5. Implications for Future Research

The disparities in professional and emotional support in the home environment between caregivers of children with ASD and non-CSHCN need further exploration. Parents of children with ASD reported different levels of poverty and different types of insurance than those of non-CSHCN. The financial advantage of parents of non-CSHCN might have allowed extended professional support. Further, the literature describes the stress that parents experience while raising a child with ASD, but little is known about how this stress extends to the home environment for other family members and how they cope.

Although in the current study parents of children with ASD or non-CSHCN did not report different levels of support from a place of worship, other studies noted that faith and spirituality offered significant coping strategies among parents caring for children with a disability such as ASD [27,62,63]. Spirituality has been found to be helpful to parents in viewing their children with ASD through a positive lens [63], and it was associated with lower parental anxiety symptoms [64]. One study of spirituality-based online support groups also proved to be effective for mothers of children with ASD in alleviating parental stress, and building self-efficacy and resilience [65]. Spiritual connection also has been found to be a protective factor against depressive symptoms in parents of CSHCN [66]. The evidence signals a need for future study regarding spiritual support in the home environment as a resilience-building resource for parents of children with ASD, and to provide holistic emotional support.

Exploring training and education for health professionals who care for families with children with ASD is another avenue for research. This provides two-fold opportunities: one for education and curriculum design in training to examine the health impacts of the home environment for families with CSHCN, as well as opportunities to provide evidence-based strategies to improve the quality of parent–child interactions.

## 6. Conclusions

In summary, the findings of this study highlighted the disparities in the home environment regarding equity: accessibility and sources of parental emotional support. The data addressed the home environment and parent–child interactions between children with ASD and non-CSHCN aged 3–5 years old across various severity levels of ASD. Such understanding of the significance of well-being in the home environment allows for further exploration of targeted emotional support and educational interventions to promote healthy families.

## Figures and Tables

**Table 1:** Frequency distributions of child and family sociodemographic characteristics by children’s ASD (vs. non-CSHCN) status.

Characteristic	Entire Sample *N* = 10,041	Children with No Special Health Care Needs (Non-CSHCN) *N* = 9798	Children with ASD *N* = 243
Children	n (%)	n (%)	n (%)
Ages			
Age 3	3403 (34%)	3330 (34%)	73 (30%)
Age 4	3358 (33%)	3278 (33%)	80 (33%)
Age 5	3280 (33%)	3190 (33%)	90 (37%)
Gender			
Male	5037 (50%)	4856 (49.56%)	181 (74.49%)
Female	5004 (50%)	4942 (50.44%)	62 (25.51%)
Race/Ethnicity			
Hispanic	1332 (13%)	1286 (13.13%)	46 (19%)
White, Non-Hispanic	6774 (67%)	6632 (67.69%)	142 (58%)
Black, Non-Hispanic	573 (6%)	553 (5.64%)	20 (8%)
Other, Non-Hispanic	1362 (14%)	1327 (13.54%)	35 (14%)
General Health			
Excellent	7841 (78%)	7748 (79%)	93 (38%)
Very good	1875 (19%)	1785 (18%)	90 (37%)
Good	275 (3%)	234 (2%)	41 (17%)
Fair	30 (0.30%)	12 (0.12%)	18 (7%)
Poor	1 (0.01%)	1 (0.01%)	0 (0)
Insurance			
Public health insurance only	2049 (21%)	1955 (20.19%)	94 (39.83%)
Private health insurance only	7067 (71%)	6974 (72.04%)	93 (39.41%)
Public and private insurance	336 (3%)	296 (3.06%)	40 (16.95%)
Uninsured	465 (5%)	456 (4.71%)	9 (3.81%)
Household			
Federal Poverty Level			
0–99%	1170 (12%)	1112 (11.35%)	58 (23.87%)
100–199%	1708 (17%)	1655 (16.89%)	53 (21.81%)
200–399%	3143 (31%)	3075 (31.38%)	68 (27.98%)
400% or greater	4020 (40%)	3956 (40.38%)	64 (25.34%)
Highest Education			
Less than high school	209 (2%)	199 (2.03%)	10 (4.12%)
High school or GED	1156 (12%)	1115 (11.38%)	41 (16.87%)
Some college or technical school	2106 (21%)	2022 (20.64%)	84 (34.57%)
College degree or higher	6570 (65%)	6462 (65.95%)	108 (44.44%)

**Table 2:** Means and standard deviations of child and family sociodemographic characteristics by children’s ASD (vs. non-CSHCN) status.

Characteristics	Non-CSHCN (Mean, SD)	Children with ASD			
		Total (Mean, SD)	Mild (Mean, SD)	Moderate (Mean, SD)	Severe (Mean, SD)
Child: age	3.99, 0.82	4.07, 0.82	4.07, 0.82	4.06, 0.84	4.08, 0.78
Child: sex ^a^	1.50, 0.50	1.26, 0.44	1.25, 0.43	1.29, 0.45	1.17, 0.38
Child: race/ethnicity ^b^	0.68, 0.47	0.58, 0.49	0.57, 0.50	0.60, 0.49	0.58, 0.50
Child: general health ^c^	4.77, 0.49	4.07, 0.92	4.30, 0.83	3.90, 0.93	3.92, 1.06
Child: insurance ^d^	0.25, 0.43	0.44, 0.50	0.35, 0.48	0.47, 0.50	0.61, 0.50
Household: FPL level ^e^	0.40, 0.49	0.26, 0.44	0.35, 0.48	0.22, 0.41	0.17, 0.38
Parent/caregiver: highest education ^f^	0.87, 0.34	0.79, 0.41	0.82, 0.38	0.78, 0.41	0.75, 0.44

^a^. Sex: 1 = male, 2 = female. ^b^. Race/ethnicity: 1 = White, Non-Hispanic, 0 = Others; ^c^. General health: 5 = excellent, 1 = poor; ^d^. Insurance: 1 = no private insurance or uninsured, 0 = have private insurance; ^e^. Household FPL level: 1 = 400% FPL or greater, 0 = below 400% FPL; ^f^. Parent highest education: 1 = with college degree or higher, 0 = do not have college degree.

**Table 3:** Parental Emotional Support Accessibility (PESA) and sources by Children’s ASD (vs. non-CSHCN) status.

Sources	Non-CSHCN (Mean, SD)	Children with ASD			
		Total (Mean, SD)	Mild (Mean, SD)	Moderate (Mean, SD)	Severe (Mean, SD)
Health professionals ^a^	0.33, 0.55	0.56, 0.71	0.42, 0.67	0.68, 0.72	0.41, 0.67
Advocacy and peer groups ^b^	0.17, 0.42	0.35, 0.66	0.27, 0.59	0.42, 0.72	0.27, 0.63
Faith community ^c^	0.23, 0.42	0.22, 0.41	0.23, 0.42	0.22, 0.41	0.18, 0.39
Family and friends ^d^	1.57, 0.01	1.42, 0.05	1.38, 0.85	1.53, 0.73	1.09, 0.92
Total PESA ^e^	2.29, 1.41	2.54, 1.78	2.29, 1.69	2.84, 1.73	1.95, 2.15

^a^. Health professionals: range = 0–2. ^b^. Advocacy and peer groups: range = 0–2. ^c^. Faith community: range = 0–1. ^d^. Family and friends: range = 0–2. ^e^. Total PESA was computed by summing the scores of “health professionals”, “advocacy and peer groups”, “faith community”, and “family and friends”. Higher scores indicate higher levels of overall accessibility to parental emotional support.

**Table 4:** Positive parent–child interactions (PCI) by children’s ASD (vs. non-CSHCN) status.

Indicators	Non-CSHCN (Mean, SD)	Children with ASD			
		Total (Mean, SD)	Mild (Mean, SD)	Moderate (Mean, SD)	Severe (Mean, SD)
Read to the child ^a^	3.07, 0.94	2.95, 0.99	2.99, 0.96	2.91, 1.00	3.04, 1.10
Tell stories and/or sing to the child ^b^	3.09, 0.93	3.20, 0.94	3.28, 0.94	3.14, 0.94	3.26, 0.92
Family sharing meals together ^c^	3.41, 0.78	3.22, 0.96	3.29, 0.95	3.15, 0.98	3.35, 0.88
Total PCI ^d^	9.57, 1.95	9.39, 2.17	9.59, 2.17	9.24, 2.13	9.65, 2.23

^a^. Read to the child: 1= “0 days”, 4 = “every day”. ^b^. Tell stories and/or sing to the child: 1= “0 days”, 4 = “every day”. ^c^. Family sharing meals together: 1= “0 days”, 4 = “every day”. ^d^. Total PCI was computed by summing the scores of “read to the child”, “tell stories and/or sing to the child”, and “family sharing meals together.” Higher scores indicate more positive levels of parent–child interactions at home.

## Data Availability

Publicly available datasets were analyzed in this study. This data can be requested here: https://www.childhealthdata.org/help/dataset. Accessed on 14 September 2022.
